# Prevalence of Methicillin-Resistant Staphylococcus aureus in Saudi Arabia: A Systematic Review and Meta-Analysis

**DOI:** 10.7759/cureus.70230

**Published:** 2024-09-26

**Authors:** Taif K Alanzi, Osama A Alhazmi, Khalid Alanezi, Waad M Alammari, Arwa A Alrwily, Muath M Alshammari, Reem Albuhairan

**Affiliations:** 1 Laboratory Medicine, College of Applied Medical Sciences, Al Jouf University, Sakaka, SAU; 2 Internal Medicine, Faculty of Medicine, Jazan University, Jazan, SAU; 3 College of Medicine, King Saud University, Riyadh, SAU; 4 Community Health Sciences, University of Tabuk, Tabuk, SAU; 5 College of Medicine, Al Jouf University, Sakaka, SAU; 6 College of Medicine, Hail University, Hail, SAU; 7 College of Medicine, King Saud bin Abdulaziz University for Health Sciences, Riyadh, SAU

**Keywords:** antibacterial resistance, healthcare-associated infections in saudi arabia, hospital-acquired mrsa, methicillin-resistant staphylococcus aureus (mrsa), mrsa epidemiology in saudi hospitals, mrsa prevalence, mrsa systematic review in saudi arabia, multidrug-resistant bacteria, saudi arabia, systemic review and meta-analysis

## Abstract

Methicillin-resistant *Staphylococcus aureus* (MRSA) is a public health concern due to its capacity to build biofilms and acquire drug resistance. The purpose of this systematic review and meta-analysis is to analyze the prevalence of MRSA in Saudi Arabia and look into its resistance to various antibiotics. A comprehensive systematic search for key scientific databases from 1990 to June 2024 was carried out using predetermined keywords. MRSA prevalence rates were examined in studies that met the inclusion criteria, with an emphasis on clinical and community settings. The MedCalc software was used to conduct statistical analysis. The meta-analysis includes 24 trials with 16,646 samples. The overall prevalence of MRSA was discovered to be 8.601% (fixed effects) and 17.027% (random effects), with confidence ranges of 8.179-9.037 and 12.503-22.091, respectively. The research showed substantial antibiotic resistance, notably to β-lactams, whereas vancomycin remained effective against most MRSA strains. The data show an alarming incidence of MRSA in hospital settings, as well as significant resistance to numerous antibiotic classes, emphasizing the importance of improved infection control strategies and sensible antibiotic usage. Geographic differences in resistance patterns point to regional factors impacting MRSA spread and resistance. This analysis highlights the significant prevalence of MRSA in Saudi clinical settings, as well as the complexities of its resistance profiles. Continued observation and study into the genetic mechanisms that contribute to resistance are critical for successful MRSA infection management and prevention.

## Introduction and background

*Staphylococcus aureus *causes a wide range of acute and chronic infections [1]. One significant virulence component is the ability to form robust biofilms on diverse surfaces [2]. This bacterium can create toxins that cause invasive disorders like skin infections, abscesses, wound infections, pneumonia, and endocarditis. Furthermore, it creates enterotoxins, which can cause food poisoning [3,4]. As with any infection, particular symptoms such as red, painful, pus-filled pimples, fever, and swollen skin are typical [5]. *S. aureus* has a variety of antibiotic resistance mechanisms, including methicillin resistance, which is generally referred to as methicillin-resistant *Staphylococcus aureus* (MRSA) [6]. MRSA is presently the most frequently detected antibiotic-resistant pathogen in numerous regions across the globe [7]. Recent findings indicate that domestic animals, such as food animals, can act as reservoirs and carriers of MRSA and that transmission between different host species is a possibility [8]. Recent years have seen changes in the epidemiology of MRSA. Concerns about the prevalence of MRSA in associated food items have grown significantly since the bacteria first appeared in animals used to produce food [9]. Depending on the nation and the animal's place of origin, the frequency of MRSA in food varies significantly [10]. MRSA has been found in a variety of meat products marketed in retail settings by many investigations [11]. In general, raw meats (including pig, beef, lamb, chicken, turkey, and even rabbit on one occasion), dairy items (like milk and cheese), and pancakes were the food groups where MRSA was detected. The research that is currently accessible indicates that poultry was more prevalent in the Netherlands and Denmark, whereas pork was most contaminated in the United States and Canada [12-15]. MRSA isolates were further classified as healthcare-associated (HA)-MRSA or community-acquired (CA)-MRSA, suggesting that food handlers are a likely source of the bacteria [13-16]. In other studies, livestock-associated (LA)-MRSA strains were the main isolates, indicating an animal origin of contamination [8].

MRSA strains were first detected in humans in the 1960s and have now become prominent nosocomial diseases, with diverse HA-MRSA clones spreading internationally. Before the 1980s, MRSA infections were limited to hospitals and primarily impacted immunocompromised people. However, in the late 1980s and early 1990s, MRSA began to emerge as a major cause of CA-MRSA infections, first in the Oceania region and then globally [17,18]. CA-MRSA and HA-MRSA have unique characteristics, with CA-MRSA exhibiting a more aggressive phenotype. They usually express the Panton-Valentine leukocidin toxin, which has been associated with severe skin infections. Interestingly, these strains have been shown to affect people who do not have the usual risk factors for MRSA infections [19,20]. Moreover, there is now a growing concern as CA-MRSA lineages are increasingly responsible for nosocomial infections, blurring the lines between CA-MRSA and HA-MRSA strains [20].

In the past 50 years, antibiotics have played a major role in decreasing mortality rates. However, it is important to acknowledge that bacteria have shown a remarkable ability to develop resistance against the majority of antimicrobial agents currently in use [7]. The methicillin resistance observed in *S. aureus* is due to the presence of the mecA gene located within the mobile genetic elements of MRSA strains. This gene encodes penicillin-binding protein 2a, which exhibits reduced affinity for β-lactam antibiotics, enabling MRSA strains to thrive in varying concentrations of these antimicrobial agents [21].

In Taif, Saudi Arabia, a research study was conducted on 93 inmates and 19 prison employees to assess methicillin resistance. The standard cefoxitin disk diffusion method and the oxacillin screen agar procedure were used for evaluation. The results revealed a high MRSA colonization rate among the inmates (24.7%) and employees (15.8%). Factors such as the long duration of residence in the correctional institution and poor hand hygiene were identified as significant risk factors for this colonization [22].

In another study, 188 nasal swabs were obtained from individuals residing in Riyadh, with 105 being males and 83 females. Out of the total nasal swabs collected, the MRSA colonization rate was found to be 9.04%, with 11 females (5.85%) and six males (5.71%) testing positive. Approximately 47% of the MRSA strains showed multidrug resistance (MDR) by exhibiting acquired resistance to β-lactam, macrolide, and aminoglycoside antibiotics [2]. In 2010, it was estimated that MRSA caused illness in over 150,000 individuals annually in healthcare facilities in the European Union [23]. Recent statistics indicate that invasive HA-MRSA infections have decreased, while CA-MRSA infections are still on the rise [24]. A few distinct lineages are linked to pandemic clones, and MRSA isolates exhibit an impressive clonal structure [25]. MRSA is currently found all over the world and poses a serious threat to human health because of its intricate epidemiology and capacity to develop new resistance mechanisms to antibiotics [12]. In 2006, the Association for Professionals in Infection Control and Epidemiology, Inc. (APIC) carried out a nationwide survey on MRSA prevalence, which revealed that 46 out of 1,000 patients in the United States had either MRSA infection or colonization [26]. The Canadian Nosocomial Infection Surveillance Program, which involved 47 sentinel Canadian hospitals, reported an incidence of MRSA in 2007 of 8.62 cases (2.57 infection and 5.87 colonization) per 1,000 patient admissions and 11.63 cases (3.47 infection and 7.92 colonization) per 10,000 patient days [27]. Furthermore, in 2007, there were 0.2 MRSA bloodstream infections per 100,000 patient days in Sweden and 2.4 in Portugal in Europe [28]. MRSA nasal carriage in colonized individuals can serve as an internal reservoir for clinical infections or as a means of spreading cross-colonization within the community [29]. Researchers have found that traditional susceptibility testing methods, such as the diffusion disk technique, may not always be accurate in detecting methicillin-resistant staphylococci [30].

Forty-three patients (11%) with an average age of 10.4±7.2 years who underwent respiratory sample testing out of 385 patients tested positive for MRSA in a Saudi Arabian research that examined the prevalence of MRSA in cystic fibrosis (CF) patients. Twenty-two (51%) of these 43 patients were given different treatment regimens: 13 patients (59%), five patients (23%), one patient (5%), and three patients (14%) all received nasal Bactroban; five patients received a combination of nasal Bactroban, oral vancomycin, and rifampicin for two weeks. Eight (36%) of the 22 treated individuals were able to completely eliminate MRSA. But after 3-6 months, six individuals (or 27%) developed a recurrence of MRSA, and five patients (23%) continued to have MRSA colonization for 2-4 years despite following an eradication protocol. Tragically, 12 out of the 43 patients (28%) with MRSA infection passed away [31]. One hundred and six MRSA isolates from infection (51) and carrier colonization sites (55) were genetically characterized using SCCmec and MLST genotyping methods, as well as the detection of PVL, TSST-1, and enterotoxins [32]. Despite the resistance MRSA showed against many antibiotics, vancomycin and linezolid are still showing good results when used in patients with MRSA [21]. Unfortunately, we noticed an insufficient number of studies looking into the prevalence of MRSA in Saudi Arabia. Hence, this review aims to assess the prevalence of MRSA in Saudi Arabia.

## Review

Methodology

Strategy for Scientific Database Search

Two authors were tasked to conduct a comprehensive search of the main scientific databases for data. The Web of Science, PubMed/MEDLINE, ProQuest, and Google Scholar were the databases used for advanced data searches. Every study was selected and reviewed. There were no other criteria used, such as patient age, gender, or demographic group. To avoid confusion and data loss, the search phrases for the data were established. The selected keywords included "Saudi Arabia" and "Middle East," along with the terms "methicillin-resistant *Staphylococcus aureus*" (MeSH) OR "MRSA" OR "multidrug-resistant *Staphylococcus aureus*" (all fields). Data was taken out between June 2024 and 1990.

Selection Criteria for Extracted Records

Any scientific work carried out in Saudi Arabia between 1990 and June 2024 that documented the prevalence of MRSA in either gender meets the inclusion requirements for records. The authors chose each study twice separately in order to prevent data bias. To carry out this study, all articles from all demographic groups were facilitated.

Non-scientific, incomplete, or inconclusive conference abstracts or posters, reviews, letters to the editor, case reports, and non-English research were all omitted from the data.

Primary Outcome Measure of the Study

The assessment of the Saudi population's MRSA prevalence is the primary outcome measure of the study.

Selection of Studies

Every extracted data was carefully reviewed by each author, who selected every item based on the inclusion criteria. The research team reached an agreement on the chosen studies and used consensus to clear up any ambiguity.

Data Extraction

Data extraction was performed in a duplicate manner independently to avoid any data loss or risk of bias in accordance with the Cochrane tool [33].

Methodology Statement and Data Presentation

**Figure 1:**
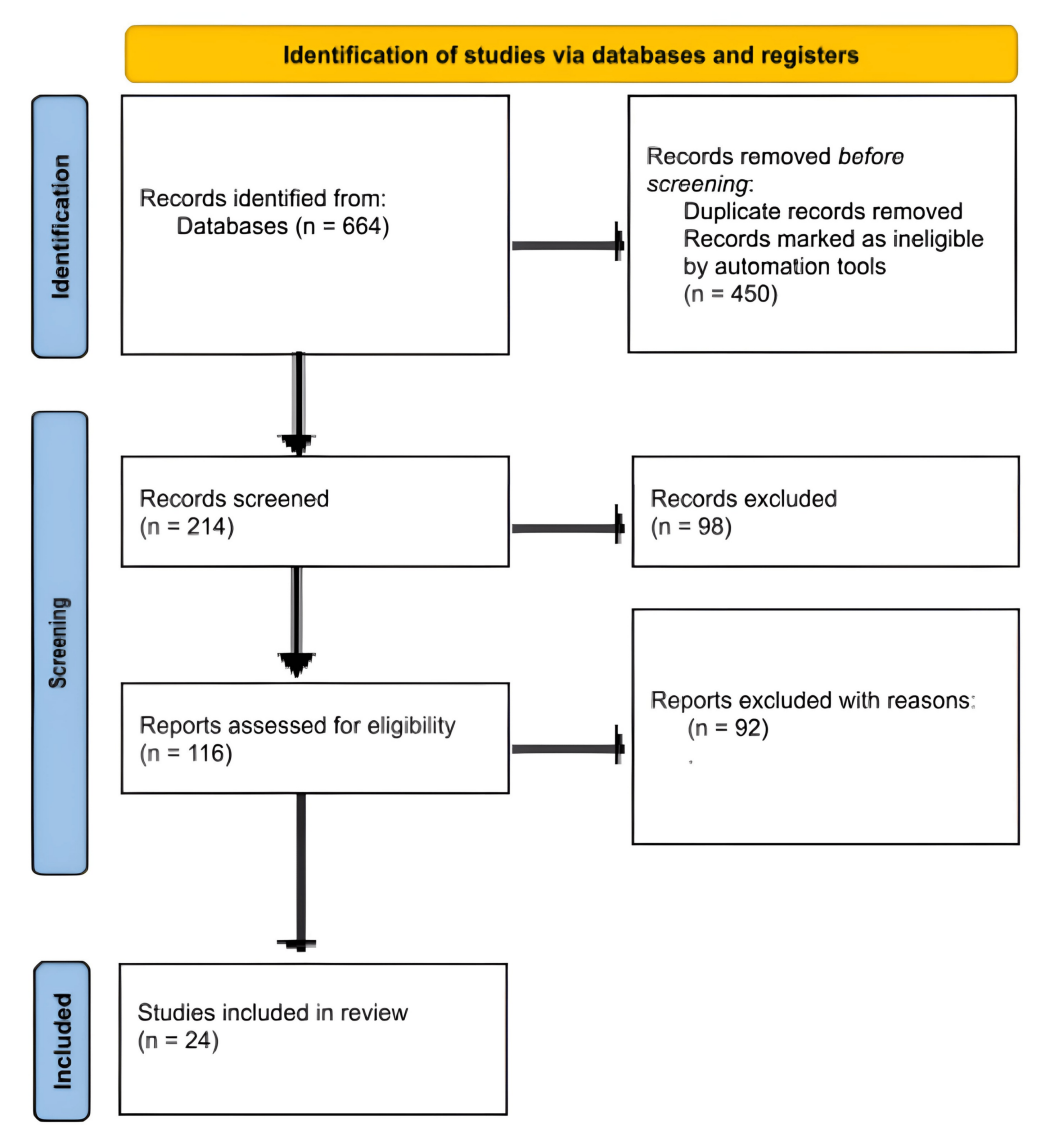
Flowchart of the study selection process

After the data were retrieved and processed in line with the inclusion criteria, they were carefully reviewed to make sure they were eligible and selected. The methodology statement and data presentation for the systematic review and meta-analysis (SR and MA) were derived from the Preferred Reporting Items for Systematic Reviews and Meta-Analyses (PRISMA) [34], as seen in Figure 1.

Statistical Analysis

The statistical analysis program MedCalc Version 22.030 (https://www.medcalc.org/) was utilized to analyze the study's parameters.

A comprehensive review of all the identified and chosen studies is outlined in Table 1.

**Table 1:** Characteristics of the selected studies MRSA: methicillin-resistant *Staphylococcus aureus*; SSI: surgical site infection; MNGHA: Ministry of National Guard Health Affairs; KAMC-R: King Abdulaziz Medical City-Riyadh; KAMC-J: King Abdulaziz Medical City-Jeddah; KAH: King Abdulaziz Hospital-Al-Ahsa; IABFH: Imam Abdulrahman Bin Faisal Hospital-Dammam

S. no.	Author, year, and reference no.	Study population	Gender	Total no. of the study population	MRSA prevalence (%)
1	Alghizzi and Shami, 2021 [5]	Milk and cheese samples in Riyadh	-	100	72.9%
2	Alzahrani et al., 2021 [22]	Inmates and correctional officers from Taif	Males	112	24.7% among inmates; 15.8% among officers
3	Banjar et al., 2020 [31]	Cystic fibrosis patients in a tertiary hospital in Saudi Arabia	Both	385	11%
4	Khan et al., 2021 [35]	Tertiary eye hospital in Riyadh	Both	430	11.7%
5	El-Deeb et al., 2018 [36]	Goats from the east province of Saudi Arabia	Both	1010	2%
6	Al Musawi et al., 2022 [37]	Cases of positive MRSA from the University King Fahad Hospital	Both	1338	Increasing trend from 5.2% to 14.5%
7	Zakai, 2015 [38]	Medical students at King Abdulaziz University	Both	150	6.7%
8	El Amin and Faidah, 2012 [39]	Medical records of *S. aureus*-infected patients	Both	186	39.5%
9	Almutairi et al., 2024 [40]	Pediatric (<18 years) and maternal patients with *S. aureus* infection	Both	152	45.4%
10	Alaklobi et al., 2015 [41]	Pediatric emergency department patients in King Saud Medical City, Riyadh	Both	830	4.6%
11	Iyer et al., 2014 [42]	Healthcare workers in Jeddah, Saudi Arabia	Both	150 participants: 100 case group and 50 control group	73% of the case group
12	Abo-Amer et al., 2022 [43]	*S. aureus*-infected patients in Taif, Saudi Arabia	Both	72 samples of patients diagnosed with *S. aureus*	49%
13	Alarjani et al., 2021 [44]	Neonates associated with sepsis disease admitted to medical college hospitals in Riyadh, Saudi Arabia	Both	151	26.9%
14	Madani, 2002 [45]	King Abdulaziz Medical City in Riyadh	Both	292	38%
15	Ghanem et al., 2013 [46]	Patients managed by the palliative care service of King Fahad Specialist Hospital-Dammam	Both	289	8.3%
16	Alhussaini, 2016 [47]	Patients admitted to Shaqra General Hospital, Shaqra	Both	220	21.82%
17	Alhunaif et al., 2021 [48]	Patients with *S. aureus* bacteremia admitted to King Abdulaziz Medical City, Riyadh	Both	633	29.1%
18	Albarrag et al., 2020 [49]	Residents and nursing staff in nursing homes in Riyadh	Both	188	9.04%
19	Alkuraythi et al., 2023 [50]	84 non-duplicates clinically identified as *S. aureus* routine cultures were obtained from inpatients at the bacteriology department in the Riyadh Regional Laboratory and Blood Bank and 250 retail meat samples including camel, beef, chicken, fish, and lamb were collected from various meat retailers throughout Riyadh, Saudi Arabia	-	76 staphylococci isolates	21%
20	Panhotra et al., 2005 [51]	Newly admitted patients in King Fahad Hospital and Tertiary Care Center, Al Hofuf	Both	600	19.1%
21	Al-Tawfiq, 2006 [52]	Patients from Saudi Aramco Medical Services Organization, Dhahran	Both	5162	6%
22	Bukharie and Abdelhadi, 2001 [53]	Patients hospitalized in King Fahad Hospital of the University, Al-Khobar	Both	420 patients in 1998; 448 patients in 1999; 450 patients in 2000 (a total of 1318)	5% in 1998; 9.8% in 1999; 10% in 2000 (8.35% of the total)
23	El-Saed et al., 2020 [54]	Patients with SSI following surgical procedures in the MNGHA hospitals: KAMC-R, KAMC-J, KAH, and IABFH	Both	403 SSI events	*S. aureus* was 89.6% and MRSA was 33.3% of these cases
24	Shibl et al., 1994 [55]	Patients’ samples from five Riyadh hospitals	Both	2300 (2000 *S. aureus* and 300 *S. epidermidis*)	5% of *S. aureus* and 30% of *S. epidermidis*

Result

A total of 24 were eligible for the meta-analysis, describing a total sample size of 16646. The studies were conducted from 1990 to June 2024. When categorizing the publishing dates for the studies used to decades, one study was published between 1990 and 2000 [55], four studies were published between 2001 and 2010 [46,51-53], 10 studies were published between 2011 and 2020 [31,35,37,39,40,42,43,47,48,54], and nine studies were published from 2021 till the date of the current study [5,22,36,38,41,44,45,49,50]. The meta-analysis shown in Table 2 demonstrated that the prevalence of MRSA was 8.601% (fixed effects) and 17.027% (random effects), with a 95% confidence interval (CI) of 8.179-9.037% and 12.503-22.091%, respectively.

**Table 2:** Meta-analysis of the prevalence of MRSA in Saudi Arabia MRSA: methicillin-resistant *Staphylococcus aureus*

Study	Sample size	Proportion (%)	95% CI	Weight (%)	
				Fixed	Random
Alghizzi and Shami, 2021 [5]	100	51.000	40.804-61.136	0.61	3.95
Alzahrani et al., 2021 [22]	172	23.837	17.682-30.917	1.04	4.11
Albarrag et al., 2020 [49]	188	9.040	5.354-14.080	1.13	4.13
Banjar et al., 2020 [31]	385	11.000	8.055-14.560	2.32	4.24
Khan et al., 2021 [35]	430	4.880	3.045-7.364	2.59	4.25
El-Deeb et al., 2018 [36]	1010	1.980	1.213-3.042	6.06	4.31
Al Musawi et al., 2022 [37]	1338	14.500	12.656-16.502	8.03	4.32
Zakai, 2015 [38]	150	18.700	12.805-25.873	0.91	4.08
El Amin and Faidah, 2012 [39]	186	39.500	32.425-46.916	1.12	4.13
Almutairi et al., 2024 [40]	152	45.400	37.316-53.667	0.92	4.08
Alaklobi et al., 2015 [41]	830	4.600	3.278-6.255	4.99	4.30
Iyer et al., 2014 [42]	100	73.000	63.198-81.393	0.61	3.95
Abo-Amer et al., 2022 [43]	72	47.000	35.123-59.131	0.44	3.82
Alarjani et al., 2021 [44]	151	3.300	1.077-7.543	0.91	4.08
Madani, 2002 [45]	292	38.000	32.409-43.838	1.76	4.21
Ghanem et al., 2013 [46]	289	8.300	5.390-12.099	1.74	4.21
Alhussaini, 2016 [47]	220	21.820	16.549-27.868	1.33	4.16
Alhunaif et al., 2021 [48]	633	29.100	25.587-32.809	3.80	4.28
Alkuraythi et al., 2023 [50]	76	14.470	7.450-24.419	0.46	3.84
Panhotra et al., 2005 [51]	600	1.100	0.429-2.299	3.61	4.28
Al-Tawfiq, 2006 [52]	5162	6.000	5.367-6.683	30.97	4.34
Bukharie and Abdelhadi, 2001 [53]	1318	9.860	8.303-11.598	7.91	4.32
El-Saed et al., 2020 [54]	492	6.900	4.824-9.511	2.96	4.27
Shibl et al., 1994 [55]	2300	4.340	3.544-5.255	13.80	4.33
Total (fixed effects)	16646	8.601	8.179-9.037	100.00	100.00
Total (random effects)	16646	17.027	12.503-22.091	100.00	100.00

**Figure 2:**
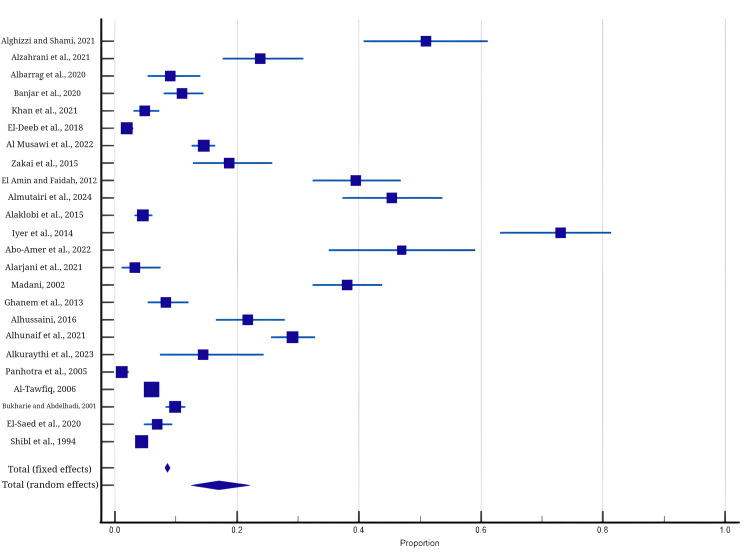
Proportion meta-analysis forest plot of all included studies References: Alghizzi and Shami, 2021 [5], Alzahrani et al., 2021 [22], Albarrag et al., 2020 [49], Banjar et al., 2020 [31], Khan et al., 2021 [35], El-Deeb et al., 2018 [36], Al Musawi et al., 2022 [37], Zakai, 2015 [38], El Amin and Faidah, 2012 [39], Almutairi et al., 2024 [40], Alaklobi et al., 2015 [41], Iyer et al., 2014 [42], Abo-Amer et al., 2022 [43], Alarjani et al., 2021 [44], Madani, 2002 [45], Ghanem et al., 2013 [46], Alhussaini, 2016 [47], Alhunaif et al., 2021 [48], Alkuraythi et al., 2023 [50], Panhotra et al., 2005 [51], Al-Tawfiq, 2006 [52], Bukharie and Abdelhadi, 2001 [53], El-Saed et al., 2020 [54], Shibl et al., 1994 [55]

Figure 2 shows the forest plot of all included studies in this meta-analysis.

This study investigates the prevalence of MRSA in clinical settings and its resistance patterns to various antibiotics. The findings indicate a significant presence of MRSA strains, particularly within hospital environments, reinforcing the growing concern regarding nosocomial infections. These results align with previous studies that have reported a similar prevalence rate, underscoring the persistent threat posed by MRSA.

Discussion

Antibiotic Resistance

The study found significant resistance to many medications, particularly β-lactams. This is consistent with the literature, which describes MRSA as resistant to methicillin and other penicillin-related antibiotics. What is particularly worrying is the observed resistance to other antibiotic classes, such as fluoroquinolones and aminoglycosides. This underlines MRSA strains' adaptive capabilities, which may complicate treatment options and increase morbidity and fatality rates.

In comparison to previous research, our study's antibiotic resistance patterns are consistent with global trends. However, geographical variances were found, particularly in antibiotic resistance, which could be attributable to changes in antibiotic-prescribing methods, infection control strategies, and local MRSA strain variety.

Effective Antibiotics 

Certain medications, such as vancomycin and linezolid, remained effective against MRSA strains. This outcome is reassuring, as vancomycin is still one of the last resorts for treating MRSA infections. Nonetheless, the advent of vancomycin-resistant *S. aureus *(VRSA) in some places necessitates continued surveillance and the prudent administration of this drug to prevent future resistance development.

Limitation

One limitation of this study is that it is based on samples from a single geographic region, which may not accurately reflect global MRSA resistance trends. Future research should aim to use a more diverse sample pool to increase the generalizability of the findings. Furthermore, molecular typing of MRSA strains might reveal more about the genetic processes underlying their resistance patterns, which was outside the scope of our research.

## Conclusions

This study emphasizes the high frequency of MRSA in clinical settings and its complicated resistance to different antibiotics. The findings emphasize the need for careful infection control measures and the wise use of antibiotics in combating the spread of MRSA. While vancomycin and linezolid remain effective therapeutic options, the possibility of resistance to these antibiotics necessitates ongoing monitoring.

Further research is needed to investigate the genetic causes of resistance and create novel ways for effectively managing MRSA infections. The continuous global effort to track MRSA developments and resistance patterns is critical for guiding clinical practice and preserving public health.
